# Making sense of undergraduate nursing students’ learning about leadership: a qualitative study

**DOI:** 10.1186/s12909-026-10848-w

**Published:** 2026-11-13

**Authors:** Ulrika Schüldt Håård, Ann Jacobsson, Per J. Palmgren, Mia von Knorring

**Affiliations:** 1 Department of Learning, Informatics, Management and Ethics, Karolinska Institutet, Stockholm, Sweden; 2 Department of Nursing, Umeå University, Umeå, Sweden

**Keywords:** Leadership learning, Nursing, Undergraduate education, Written reflections, Thematic analysis

## Abstract

**Background:**

Leadership is a core competence for registered nurses and included in undergraduate nursing education (Bachelor of Science in Nursing, BSc Nursing). Yet newly graduated nurses report that they are unprepared to lead. Understanding how students learn leadership is essential for strengthening their preparation for future professional roles. This study explored how nursing students’ approach and make sense of their learning about leadership within the context of undergraduate education.

**Method:**

The study used an interpretative qualitative design to conduct a retrospective analysis of written reflections from 247 nursing students who had completed a leadership course during their second year of study. This included students who were in-person learners and online learners. Guided by reflective thematic analysis and informed by transformative learning theory, both manifest and latent content of the data was considered in the analysis.

**Results:**

The results show that students’ learning is shaped by meaning-making and reflective engagement, drawing on past experiences, present course activities, and anticipated future professional roles. Students’ learning strategies ranged from individual reflection to collaborative engagement with peers. Three interrelated themes that show how this was done were identified in the analysis: using group work to understand leadership; applying knowledge in meaningful context to foster leadership insight and reflection, and personal development as drivers of leadership growth. Together, these themes demonstrate how students internalise leadership as part of their emerging professional identity.

**Conclusion:**

Nursing students’ learning about leadership can be understood as a process of meaning-making shaped by their approaches to learning and the integration of experiences from clinical and academic contexts. Leadership learning involves both collaborative and individual activities, supporting nursing students in developing the capacity to lead others and themselves. Leadership education should therefore attend to how nursing students make sense of leadership by building on their experiences, integrating theory and practice, and providing opportunities for group learning alongside individual reflection. Positioning leadership education in the middle of the undergraduate programme appears appropriate, enabling students to draw on clinical experience while continuing to develop leadership skills during subsequent placements prior to graduation.

## Background

Leadership is a key factor in addressing the challenges facing the healthcare sector [1] and is considered a core competence for registered nurses (RNs) [2–4]. Yet, research [5, 6] indicates that newly graduated nurses often feel insecure and unprepared to lead. Few studies have explored how nursing students learn about leadership during their undergraduate studies. A deeper understanding of this process could inform the development of appropriate educational support to enhance student learning and prepare students for their leadership roles as registered nurses (RNs).

The need to support leadership in the nursing workforce – both in senior leading positions and among newly graduates – has been emphasized by the World Health Organisation [2]. When identifying competencies shared across countries, Wit et al. [4] proposed ten ‘generic competency domains for RNs. One of these domains is leadership, encompassing competencies such as: *professional leadership*,* lead and managing care*,* lead and influence others*,* demonstrate self-awareness and coordinating multidisciplinary teams* [4, p. 6].

In an often-used definition Yukl [7, p.24] describes leadership as *“the process of influencing others to understand and agree about what needs to be done and how to do it*,* and the process of facilitating individual and collective efforts to accomplish shared objectives”.* This definition encompasses key characteristics such as influencing others, adopting a facilitative role, focusing on shared goals and objectives, and reaching consensus on how to achieve them. For RNs, leadership is also embedded in their profession [8] and often referred to as “clinical leadership” [9], which incorporates the responsibility to lead, plan, organize, delegate, and supervise nursing care [3].

The transition from education to professional practice is widely recognized as a stressful period for newly graduated nurses [10]. Leadership has been identified as a particular challenge, as new graduates are expected to lead others despite not yet having fully developed their self-leadership [5]. A study by Topola and Miller [11] illustrates the complexity of leadership from the perspective of nursing students. Their analysis of focus group data revealed that, although students acknowledged that RNs are expected to lead in healthcare settings, students were not keen to do it, others view the role of RN to be as a facilitator within teams rather than leading the same. Furthermore, students questioned whether RNs had the authority to lead interdisciplinary teams due to distribution of power in health care.

Given the challenges associated with the transition to professional practice, there is ongoing debate about how best to prepare nursing students for their leadership roles during undergraduate education [12]. Scammell et al. [12] highlight the considerable variation in how leadership is integrated into undergraduate nursing programs – sometimes as standalone modules, and at other times embedded throughout the programme. Positioning leadership modules in the final year may inadvertently suggest that leadership is inherently linked to seniority, offering limited time to develop skills during education [12]. Instead, leadership should be regarded as a continuous process that evolves over time [13]. Early exposure to situations where leadership can be practiced allows students to begin reflecting on their self-image as leaders from the outset of their education [14]. According to James et al. [15], leadership skills occur at the intersection of nursing students’ practical experiences and theoretical knowledge. Alignment between these two elements supports student learning [15, 16].

Middleton [17] highlights active learning as valuable approach for developing leadership skills, due to its emphasis on collaboration, communication, and teamwork. These skills can be further strengthened through the integration of leadership theories, which serve to deepen both knowledge and engagement [17].

In this study we are inspired by Mezirow [18], who argues that meaningful learning requires learners’ assumptions and beliefs to be challenged. This process is central to transformative learning, which emphasizes the importance of critically reflecting on such experiences, either individually or through rational discourse in groups where new perspectives can be validated. Through this transformation in perspective, learners make meaning of the activity and arrive at new, more inclusive understandings [18].

Focusing on students’ learning process offers insight into what they perceive as meaningful. According to Mezirow [18], for new knowledge to be considered meaningful and adopted, it must be integrated with pre-existing knowledge and built upon prior experiences. However, learning may develop differently depending on the context and activities in which individuals engage [19]. When studying how nursing students approach their learning about leadership, these aspects must be considered.

Given the variation in how leadership is incorporated into nursing education and newly graduated nurses’ lack of confidence in their leadership role, there is a need to explore how students themselves understand and approach their learning of leadership. The aim of this study is to explore how nursing students’ approach and make sense of their learning about leadership within the context of undergraduate education.

## Method

### Design

An interpretative qualitative design was chosen for the study as the focus lies on human experiences, perceptions, and their contextual uniqueness. This approach is grounded in the assumption that reality is not objective and fixed, but rather subjective and socially constructed which aligns with the interpretative paradigm [20].

Written texts, produced by nursing students enrolled in an undergraduate programme at a publicly funded university in Stockholm Sweden, were used as data. The students were part of a three-year programme (180 credits) leading to both a professional qualification as RNs and a Bachelor of Science in Nursing degree.

All participating students completed a leadership course during their second year of study. Although leadership-related content is integrated throughout the programme, this was the first course in the program that specifically focused on leadership. It consisted of three modules: clinical leadership, group processes and improvement work. Group work was threaded through the course, as reflected in seminars, discussions, and a group assignment.

The individual reflection assignment, which is used as data in this study, was given on the last day of the course. Notably, The leadership course did not include any clinical placement, however all students had previous experience of that from the programme. The course was offered both as a campus-based course and as a stance learning course. Both courses followed the same course syllabus, had identical assignments and were delivered multiple times per year. Notably, although distance-learning students attended the leadership course online, the course was not delivered solely through asynchronous learning, as students also participated in scheduled sessions with a lecturer. Consequently, the course had more of a blended-learning format.

The study was guided by the Consolidated Criteria for Reporting Qualitative Research (COREQ) guidelines [21], where applicable, to enhance methodological rigor and transparency.

### Participants

All students who had completed the leadership course during their previous academic year, and obtained a passing grade, were invited to participate in the study. This included students from both the campus-based and the distance learning formats who, although belonging to different cohorts, undertook the same course on their nursing degree programme respectively. The campus-based courses varied in size throughout the year, ranging from 27 to 68 students, and were offered four times annually. In addition, the distance learning version of the course was delivered twice a year, with 24 to 32 students per cohort. The fact that all campus and distance students were approached to participate in the study, along with variation in course size and term placement, contributed to diversity. As follow-up questions were not possible, we chose to include all students’ texts to maximise variation and ensure that the full breadth of student experiences was represented. The inclusion criteria were that the student had completed the leadership course and received passing grade.

In total, 248 students were contacted via email by the first author (USH), informing them about the study and inviting participation. One person declined to participate and provided no explanation of why. Thus, 247 students were included. The group consisted of 210 women and 37 men, with an age range of 21 to 63 years.

The email included information about the study, contact details, data management procedures, and the right to withdraw from the study at any time without further explanation. An opt-out alternative was used, where the students were asked to respond if they did not want to participate. Moreover, no new additional information was requested from the students; only existing material from the completed leadership course was used. The study was submitted to the Swedish Ethical Review authority who approved of the study (Dnr 2023-05632-01).

### Data collection

Pre-existing textual data was used in the form of a course assignment. This was an individual reflection submitted at the end of the course. In this task, the students were asked open questions and encouraged to reflect on their learning about leadership in the course. In the assignment questions asked were: “Describe significant events and opportunities for development during the course”, “Which learning outcomes and personal goals did you achieve through these experiences?” and “How might these competencies might be applied to situations encountered in professional practice as a nurse?”. The students were also instructed to relate these experiences to theories they had become familiar with during the course. All course assignments were submitted electronically which facilitated both access and readability. The text varied in length between 500 and 1000 words. To ensure confidentiality, all student assignments were de-identified and assigned a code prior to analysis.

### Data analysis

A reflective thematic analysis approach [22] was used to analyse data. Initially, the analysis was inductive, data-driven, and grounded in data. After initial themes had been generated, Mezirow’s transformative learning theory [18] was used to support interpretation. The theory was not used to guide coding, and earlier-coded data were not revisited As the process evolved, the analysis therefore had an abductive character, combining inductive insights with theoretical interpretation. In the analysis, attention was given not only to the manifest content but also to the latent content, the underlying meaning of the students’ experiences expressed in the texts [23]. This is in line with Braun and Clarke [24], who state that this is necessary to get a deeper understanding of what’s being studied.

We followed the six phases of Braun and Clarke [24] starting with familiarisation of data where some of the texts were initially read and discussed among the authors. During next phase with systematic coding, the additional texts were read by the first author to search for patterns and discussed with the second author to deepen interpretation in a way that moved beyond course-specific reflections and contributed to a broader understanding of leadership learning. To manage coding at this scale, both coders first jointly developed the initial coding frame on a subset of texts. The first author then coded all remaining texts while the second author reviewed a later subset to confirm the stability of the coding and emerging patterns. Hence, the final 50 texts were read to ensure that no new meaning patterns appeared. The coded data was thereafter discussed among all authors and initial themes were generated, aiming at identifying the deeper meaning in data and grasp the bigger picture. In the next step themes were further developed and reviewed followed by definition and refinement to calibrate the themes and ensure consistency. Lastly, in the written report, themes were described and supported with illustrative quotes from the students’ texts. In the text // is used to indicate text has been omitted. The edits were made only for clarity and de-identification, without changing the meaning of the quote.

## Results

In the analysis one overarching theme, *learning through meaning-making and reflective engagement* was identified which reflected the diverse ways in how students approached their learning about leadership. The students engaged in meaning-making in relation to new knowledge or behaviours by drawing on previous experiences and knowledge, current events, and aspects they considered important for their future professional role as RNs. Furthermore, they used both intra and inter approaches, aiming at individual reflection and working together with others respectively.

Within the overarching theme three themes were identified: *(1) Using group work to understand leadership*,* (2) Applying knowledge in meaningful contexts to foster leadership insight*,* and (3) Reflection and personal development as drivers of leadership growth* (see Table 1). These themes were derived from eight sub-themes and are presented below, supported by illustrative quotations.

**Table 1:** Overview showing how students approached their learning about leadership

Sub-themes	Themes	Overarching Theme
Valuing open dialogue and mutual trust in group settings	Using group work to understand leadership	Learning through meaning making and reflective engagement
Establishing shared understanding and expectations through discussion		
Exploring potential leadership roles in collaborative environments		
Integrating new knowledge with future professional role	Applying knowledge in meaningful contexts to foster leadership insight	
Connecting research to own experiences		
Using models and tools in real situations		
Critical self-reflection on behaviour and personal development	Reflection and personal development as drivers of leadership growth	
Using reflection as a means to learn with and from others		

### Using group work to understand leadership

This theme illustrates how leadership learning emerged as a socially situated and relational process, in which interaction and co-creation with peers became central to students’ meaning-making. Group work provided a context where leadership could be explored, negotiated, and enacted, allowing students to reflect on leadership not only as a theoretical concept but as a shared practice. The theme and its three sub-themes within this theme will be discussed in detail below.

#### Valuing open dialogue and mutual trust in group settings

In their texts the students described situations where they engaged in collaborative dialogue and co-construction of understanding and how this had influenced their views on leadership. The importance of gaining alternative perspectives was highlighted by the students, both in terms of hearing the views of others and becoming more aware of one’s own perspective. This was however not without complications, since it meant exposing or sometimes explaining one’s own views to the group and thereby risk being questioned. In turn this required mutual trust among group members. By working together in groups, the students became aware of the importance of creating a good conversional climate with encouragement and feedback, where all group members could be heard and listened to.

*I’ve realised it’s really good for everyone in the team to get to know each other. It helps create a better working atmosphere. No matter our different preferences*,* things run smoothly when we’re open to each other’s differences and make the most of them. That way*,* everyone grows. Having check-ins and breaks*,* chatting about other things and getting to know one another is positive for the work because it makes things more relaxed – and that makes it easier to come up with solutions to the problems and challenges the team faces. (95)*

Disagreement or getting stuck per se was not always the problem, but rather if the problems were not solved or handled in the group. On the contrary, it could support the group development and trust if the group managed to work it through.

*“What’s taught me the most [about leadership] during the course are those times when our group got stuck or simply didn’t agree with each other. Being able to stay calm and work together to get through the challenging parts has been invaluable.” (48)*.

#### Establishing shared understanding and expectations through discussion

One challenge related to leadership that was addressed by the students was how to reach an agreement on how to work together to achieve the goal for the mutual group work. Once the students had opened to one another it became clear that there were different opinions and preferences on how to work, but also regarding the level of ambition or on how to perceive the goal –for some it was just to pass the course while others wanted to go more into depth with the leadership subject. To address this the students were encouraged to devote time for discussions, not just on what to work with, but rather on how to go about doing it, how to structure their work. One of the students described this as a prerequisite for the group work being done at all:

*“Unless the course instructors specifically ask for it*,* a lot of students tend to skip this step – setting the ground rules – and just let the group develop on its own. Quite often*,* I find this leads to simmering frustration within the group because of poor meeting discipline*,* uneven workload*,* mismatched goals*,* and so on. All too often*,* it ends with everyone just wanting to get the work done as quickly as possible with minimal interaction*,* which ultimately results in fragmented learning where the only focus is on passing and moving on to the next course.” (190)*.

A way to come to a common agreement in the group was to formulate game rules together, which addressed issues as communication platforms, how to best use the strengths in the group, how to handle conflicts, leadership and goals. These served to guide the group to the task per se and in relation to each other, to ensure that group members needed to work in the same direction and shared common plans.

*By mutually setting a few game rules*,* everyone knows how to approach the task and get along with each other. It made it clear what we expected from one another and how we’d deal with any conflicts if they came up. (14)*

#### Exploring leadership roles in collaborative environments

The small size of the groups, 4–6 members, and having agreed on common game rules paved the way for collaboration, exploring new roles and knowledge in leadership. In some cases, this meant actively taking on the role as a leader for the group even though it was something that the student had avoided before, whereas other students chose to take a step back referring to their tendency to take over the leadership role.

*I told my group that one of my challenges is that I can sometimes take up too much space and take on too much responsibility. Sometimes it’s because it genuinely needs doing when others aren’t as engaged*,* but other times it just happens for no real reason. Afterwards*,* I realise I may have taken opportunities away from others. (27)*

It was up to the groups themselves to decide on how to handle the leadership in the group, resulting in groups with one appointed group leader, alternating the role among group members or leading collectively. However, having an appointed leader in the group was raised as an opportunity to learn from others when leading the group, and a valuable experience when oneself was appointed leader. Leading a group also made it possible to experiment with new leadership roles and explore different types of leadership in practice.

*What I’ve really valued is having the chance to step into a leadership role myself*,* and seeing others do the same. It’s given me a much clearer sense of the kind of leadership style that suits me*,* and the type I work best with when collaborating. (25)*

*The most interesting thing about transformational leadership was that I didn’t just learn the theory*,* but I was also able to put that knowledge into practice during the times when I led my study group. (21)*

### Applying knowledge in meaningful contexts to foster leadership insight

In this theme, the students described how they dealt with and critically engaged with new knowledge in light of prior or present experiences and future responsibilities. In doing so they linked this new knowledge to things that were familiar to them. These familiar contexts concerned both the occupational or educational sphere and sometimes private life. This allowed them not only to explore how this new knowledge fit in with what they already knew, but also in what future contexts it could be meaningful.

#### Integrating new knowledge with future professional identity

In their texts the students elaborated on what leadership might be and its potential relevance for nursing. Their reasoning evolved from a question of how leadership was manifested in RNs work, whether in a formal manager or in the professional role as nurse. This question helped them to define and formulate to themselves and others how leadership is relevant for nursing. One way in which the students made sense of leadership was reflecting on how it aligned with their expectations of an RN’s responsibilities in practice. Recalling situations from their clinical placement also helped the students in identifying how clinical leadership could be executed.

*I also feel that I’ve gained a real understanding of the nurse’s different leadership roles – things like coordinating work*,* supervising others*,* and delegating tasks. And apart from the assignments on the course that show this*,* I’ve also noticed that I can explain it much more clearly when I talk about the course with friends and family (107)*.

*I found the idea of clinical leadership and how it works fascinating. It gave me one of those ‘aha’ moments*,* thinking back to the times I was on clinical placements and saw staff who didn’t have any formal title as a manager or team leader*,* yet the rest of the team trusted them and listened to them. It’s really interesting how some people become these ‘hidden leaders”. It makes you reflect on what qualities they have and what it is that makes others want to follow them. (14)*

#### Connect research to own experiences

To make research in leadership more relatable to the students’, nursing supervision was discussed, a type of leadership they all had encountered during their clinical placements. Participating in sessions where research articles on nursing supervision were read and discussed made the students recognising situations, feelings and challenges they had experienced during clinical placements. In a sense, this served as “validation” when the experiences were shared with others and addressed by research. Furthermore, reading about supervisors’ leadership strategies led to discussions on how to lead others and how to lead oneself.

*By reflecting on the different supervisors my classmates and I had*,* while at the same time gaining more knowledge about leadership styles*,* the experiences from my clinical placements have started to make much more sense to me than they did before. I feel I’ve been able to connect those experiences to actual knowledge*,* which has helped me see them from a completely new perspective. (184)*

#### Using models and tools in real-world situations

Previous work experiences offered real situations for the students to test their new knowledge against, which could ease the learning process by providing different contexts for learning leadership. However extensive experience in practice did not necessarily mean that the students were aware of the theoretical underpinnings of leadership.

*My previous experience in leadership and improvement work is that I’ve spent several years working on organisational development and improvements within health and social care. Because of that*,* I’ve seen many different types of organisations and challenges across various areas*,* and I’ve worked with lots of different leaders and experienced a wide range of leadership styles. However*,* I haven’t really reflected much on the theoretical aspects that influence or shape leadership. (157)*

Learning leadership from theory, models and tools required processing, both to understand them per se, but also how they could be relevant when working with leadership. For instance, insights on a model for group development helped students to understand processes during group work, and that disagreement is a part of the process. Real-world examples in form of case studies were used to practice models and tools, where a fictional scenario was discussed in groups. This opened for discussions on different approaches and tools a leader could use when facing conflicts. Hence, models and tools became meaningful to the students when processed together with others, and its application became clear.

*I’ll definitely make use of what I’ve learnt […] for example in situations where conflict arises. Not only through discussing and working through a case together as a group and then suggesting solutions*,* but also through talking with each other about the tools and approaches a leader can use to both prevent and resolve conflicts. (54)*

### Reflection and personal development as drivers of leadership growth

This theme highlights how reflection contributes to students meaning making by allowing them to process how their view of leadership evolves, both in relation to their own personal development but also in relation how they reflect with others. Through this process, reflection seemed to serve as a tool for leadership.

#### Critical self-reflection on behaviour and personal development

Leadership was not a new area for the students when they embarked on the leadership course, as it had been addressed previously during their undergraduate programme. However, the extent to which the students had previously reflected on the leadership role varied considerably. For some, this proved challenging, as they were uncertain about what would be required of them, how to manage the responsibilities of being a team leader, and how to develop the necessary skills. Gaining deeper insights into leadership however, prompted the students to re-evaluate their earlier experiences and consider how they had acted in different situations, as well as how they might modify their behaviour in the future.

*“Throughout the whole programme*,* we’ve been told that nurses have a leading role — and that’s actually been my biggest worry. I’ve often asked myself*,* ‘How am I supposed to lead a team?’ But now I know that anyone can grow into a good leader*,* as long as they’re open to it.” (235)*.

*I’ve got things I need to work on if I want to become a good leader. I still struggle quite a lot with finding practical strategies to develop my weaker sides*,* and it’s not an easy process because it’s so closely tied to your personality and identity. (190)*

In their texts the students also reasoned on what they considered valuable to develop in their leadership. Aspects as being perceptive, motivational and “practice as you teach” were mentioned as important.

*With everything we’ve read*,* listened to*,* and discussed*,* I’ve started to feel a completely new sense of wanting to take on the challenge of management — to do more courses and hopefully become a good leader in the long run. But I’ve also gained the confidence to step into the leadership role that comes with being a nurse in the everyday work*,* and to try to help create a positive working environment for my colleagues and for myself. I usually try to behave in the way I’d like others to treat me — it’s a good guiding principle — but now I’ve got more tools to help me do that. (34)*

#### Using reflection as a means to learn from and with others

Reflection was not merely regarded as an individual activity, students also approached their learning about leadership through reflecting with peers during the course, which added another type of value than learning from a teacher. It was described as a collective learning where students learned with and from each other. One example of this was a group assignment where students were to lead a seminar and discussion on a written improvement work in leadership.

*Getting the chance to lead a seminar was valuable*,* even though it didn’t feel like I was doing that much at the time. But it led to some really good discussions and reflections that you wouldn’t usually get with a standard written assignment. Hearing other students’ interpretations and thoughts gave me much more to think about than if we’d just been ‘fed’ information by a lecturer. (133)*

## Discussion

This study sought to explore how nursing students’ approach and make sense of their learning about leadership within the context of undergraduate education. Rather than identifying specific leadership competencies, this study contributes insight into how leadership learning is constructed through students’ meaning-making processes within educational contexts. The study showed that the students approached their learning about leadership through meaning making and reflective engagement. Furthermore, the analysis revealed a dynamic and reflective process in which students construct meaning through past experiences, present interactions, and imagined future roles. Taken together the findings suggest that the strategies students adopted for learning leadership ranged from intra-level personal reflection to inter level collaboration with others. Three themes illuminate different yet interconnected aspects of this process: using groupwork to understand leadership, applying knowledge in meaningful contexts to foster leadership insight, and reflection and personal development as drivers of leadership growth. Together, these themes offer insight into how students engage with and internalise leadership as part of their professional formation.

### Importance of group work to understand leadership

One of the most significant findings concerned the role of group work in understanding leadership. Teamwork is also a core competence for RNs, requiring effective communication and collaboration with other healthcare professionals, patients and relatives [3]. As such, teamwork forms an integral part of the clinical leadership that is exercised by RNs. It is therefore essential that students are given opportunities to practice these skills during their undergraduate education. Our findings indicate that group work can support the development of such competences by offering interaction and co-creation, provided that mutual trust and open dialogue are present within the group. This resonates with Mezirow [18], who highlights the encountering of alternative perspectives as a necessary precondition for learning, as it allows students to critically reflect on their own assumptions and gain a more nuanced understanding of themselves and others. Hence, critical thinking and creativity are important components in preparing for the future work as RN by stimulating to innovative and effective ways of working and avoiding routine thinking [17].

Our study shows that for groups to reach a shared understanding and establish common expectations; they needed to find ways to navigate diversity. Being randomly assigned to groups posed additional challenges, as many, students had not worked together before. However, considering the anticipated professional context, this was not necessarily a disadvantage. Rather, as Allert et al. [25] suggests, random assignment provides an opportunity to managing diversity and leveraging team competence.

To support the navigating of diversity and the learning process in the group, the educator organised students into smaller groups of 4–6 members offering clear guidance on how to approach collaboration together. Small group settings have been shown to benefit group development and productivity, with increased communication identified as a key contributing factor [26]. Hence small groups and guidance are elements which supports student learning and need to be considered, in addition to the topic under study and format for reporting results.

Given the premises for the students – a short course, newly formed group constellations, and a challenging topic, there was a risk that they would focus more on *what* to do rather than *how* to do it. This reflects what McEwan et al. [27] refer to as ‘taskwork’ rather than ‘teamwork’. To support a focus on the ´how, students were given clear guidance to discuss expectations and working methods. Our findings suggest that unless educators are actively highlighting these aspects, they risk being overlooked. However, taking time to establish shared understanding and clarifying expectations early on is a worthwhile investment, to minimise conflicts later in the process. Shared goals and mutual understanding contribute to the togetherness in the team but are also important ingredients for effective communication which has an impact on both patient safety and interprofessional collaboration [25].

Our findings show that students had taken the opportunity to explore leadership in their groups, actively choosing to take on a leadership role, but also to step aside to allow others to lead in the group and, above all, learn by seeing others lead. This supports the findings reported by Scammell et al. [12], who in their review reinforce the importance of active learning and highlights how collaborative work in groups may serve as safe space for development of leadership skills. For students to participate in discourses where they get to validate their interpretations is also an important step in their meaning-making in the learning process [18].

### Importance of applying knowledge in meaningful contexts

Our findings indicate students made sense of new knowledge in leadership by applying it in meaningful context, such as previous experience. This is in line with James et al. [15] who conclude in their study that students view of leadership, and its attributes are dependent on learning as well as previous experiences, consequently the perception of leadership varies and may carry different connotations. However, our findings also suggest that students explore how new insights of leadership resonated with experience from past, present or their envisioned future profession. In addition, students referred to a broader range of contexts, (including education and educational settings, previous workplaces, and private life) when reflecting on leadership. A contributing factor could be the wide age span in our study (21–63 years), indicating that some students had more work-life extensive experience and more exposure to observed leadership in practice over a longer period than others. To enhance student learning of leadership, educators need to consider assignments which allows students to draw upon a wide range of contexts and experiences.

Our analysis indicated that students also felt their clinical experiences as being validated and contextualized through the review and discussion of research articles. However, research has shown that newly graduated nurses often struggle with resistance from colleagues when introducing research-based improvements to nursing care [6]. Strengthen nursing ability to engage with and apply research is therefore an important step in preparing them for future roles in clinical leadership or managerial roles.

Another way of learning leadership among the students involved theory. Theoretical frameworks seemed to facilitate students’ learning by providing tools for explaining processes and clarifying constituent steps. Within group-work contexts, these frameworks for example helped students make sense of group dynamics and recognise conflict as an inherent part of group development. Furthermore, students saw how theory-based tools could be applied in case discussions related to real-life examples from clinical practice, for example, in how to manage conflicts as a nurse leader. This is consistent with Mezirow’s study [18] where he discusses the premises for transformative learning and present one of these being to actively engaging students in learning, where new ideas or concepts are related to student experiences and then critically assessed to see how they make sense together with others.

Although Mezirow [18] emphasises the value of connecting new ideas with others’ perspectives, our findings also point to boundary conditions in this process. For example, Scammell et al. [12] describe group work as consistently supportive for leadership development, yet our data show that its effectiveness depended on trust and shared expectations, which were not always present in newly formed groups. This nuance suggests that collaborative meaning‑making may be more fragile and context‑dependent than previously assumed. Similarly, James et al. [15] highlight prior experience as central for developing leadership insight, whereas our findings indicate that extensive experience did not automatically lead to deeper reflection. These observations illustrate that leadership learning may vary more across contexts and individuals than earlier studies have suggested.

### The importance of reflection

Reflection served as an important means for students in our study to engage in meaning-making, enabling them to evaluate their leadership skills, identify areas for development, and consider their own behaviour in relation to past experiences. Similar findings have been described by James et al. [15] who referred to students’ growing self-awareness and evolving perceptions of leadership as “an awakening”. According to Scammell et al. [12] this reflective process is an important step in development of self-leadership among nursing students. It involves gaining insight into oneself and preparing for leading others in clinical practice. Our findings indicate that the students engaged in reflection on clinical leadership to varying degrees. Some had begun to explore and familiarize themselves with the leadership role of RNs, while others expressed openness to future manager responsibilities. The depth of reflection also varied among some students. It is possible that students with less professional experience found deeper reflections more challenging. However, for learning to be perceived as meaningful, new information must be integrated with existing knowledge [18]. This underscores the importance of incorporating learning activities in nursing education that stimulate reflective thinking and support the development of leadership competence regardless of students’ prior experience.

Our analysis revealed that that learning leadership also involves learning *from and with others* highlighting the importance of using reflection as a pedagogical tool to support leadership development. To further strengthen leadership learning in clinical practice, structured reflection together with staff members will be valuable in helping to identify fields to improve on both individual and team level [6].

Students in our study described how they learned from fellow students, particularly through leading seminars and sharing reflections, which resulted in more meaningful and thought-provoking discussions than simply receiving information in a teacher-directed format. These findings support those by Ha & Pepin [28] which highlight the importance of not only recognizing role models in clinical settings but also acknowledging the value of peer learning. In the texts students apply different types of leadership and explore concepts as management, leadership, clinical leadership and being a manager. This could be interpreted as the students are in the process of orienting themselves and an attempt to concretize what leadership means in nursing and how it is manifested in nursing practice.

### Methodological considerations

In this study we used written student assignments as data based on the assignment’s relevance for the study’s focus and their reflective nature which provided insight into students’ experience. However, since the data consist of pre-existing texts, originally produced for another purpose, there was no opportunity to ask follow-up questions to deepen understanding. In this sense it has similarities with an open-ended question. According to La Donna et al. [29] these types of questions can only be used meaningfully for qualitative analysis when they provide sufficient length and depth, and when analysis is conducted with rigour. In our study the texts were up to 1000 words, and students were encouraged to reflect and elaborate, which allowed for analysis of “how” and “why” questions in the thematic analysis. Furthermore, the data was collected from Swedish undergraduate nursing students during their second year of study. All had experience of different clinical placements during previous courses. which had exposed them to various models of leadership. Notably, although distance-learning students attended the leadership course online, the course was not delivered solely through asynchronous learning, as students also participated in scheduled sessions with a lecturer. Consequently, the course had more of a blended-learning format. However, these aspects need to be considered for the transferability of the results to other settings.

Because the texts were produced in a graded context, their content may have been influenced in ways that reflect academic writing skills rather than authentic personal reflections. To address this, student assignments from an entire academic year were included, acknowledging that both quality and length of the texts may differ. This selection encompassed assignments from both campus-based and distance education programs, as well as varying number of students, and were all included in the leadership course. This approach provided a degree of variation that enriched the dataset. The decision to only include data from students who had passed the course could be debated, however, but since the first author has been involved in the course, we wanted to avoid power relations where students were still in a dependent relationship. To strengthen the credibility, all authors participated in the analysis and interpretation of data. Rich descriptions from students’ text were included to illustrate the finding,

Being familiar with the course context and content may both enhance and shape the interpretation of the data. This preunderstanding informed the analysis and was addressed through reflexive discussions within the research team. While only including students who had passed the course may limit perspectives from students who struggled, it was considered ethically appropriate given the authors´ involvement in the course. ​Clear communication and informed consent were essential in light of the researchers’ familiarity with the context and the use of student assignments as data.

### Implications

Our findings imply that both individual reflection and group-based activities are essential to support the development of self-leadership and the ability to lead others. For educators, this requires the need to design learning activities that foster both personal individual processing and collaborative engagement. Addressing group development dynamics and encouraging shared reflection can strengthen students’ teamwork skills and prepare them for future professional collaboration.

A common starting point in learning is to relate new knowledge to prior experiences. Given the wide age span and varying backgrounds among undergraduate students, a broad definition of context is needed to enable meaningful connections.

Teachers, supervisors, and peers play a key role by being models to the students in learning leadership and by bridging between theory and practice. Actively engaging students in activities which combine these, such as case studies, or reflective seminars, will illustrate how theory and practice benefit from each other in nursing. This could strengthen the students in their learning of leadership in academia and health care settings.

Having experience from prior clinical placement appears to enhance students´ engagement in discussions on leadership. Consequently, leadership education may be most effective when delivered during the latter half of the program, allowing students to have experienced various clinical placements and yet will be able to apply and reflect on their knowledge during training.

Further research is needed to study how leadership development is enabled and executed for new RNs over time, but also how different teaching strategies influence leadership development. This calls for in-depth qualitative studies based on semi-structured interviews or focus groups but also longitudinal studies to capture changes over time.

## Conclusion

This study shows that nursing students learning about leadership is a meaning-making process shaped by how they approach learning and integrate experiences from clinical and academic contexts. Leadership develops through both collaborative and individual learning, supporting students in making sense of leading others as well as leading themselves, which has important implications for the design and timing of leadership education within undergraduate nursing programmes.

## Data Availability

The datasets used and analysed during the current study are available from the corresponding author on reasonable request.

## Abbreviation

RN: Registered Nurse
